# Using UHPLC–MS profiling for the discovery of new sponge-derived metabolites and anthelmintic screening of the NatureBank bromotyrosine library

**DOI:** 10.3762/bjoc.18.164

**Published:** 2022-11-15

**Authors:** Sasha Hayes, Aya C Taki, Kah Yean Lum, Joseph J Byrne, Merrick G Ekins, Robin B Gasser, Rohan A Davis

**Affiliations:** 1 Griffith Institute for Drug Discovery, Griffith University, Don Young Road, Brisbane, 4111, Australia; 2 Department of Veterinary Biosciences, The University of Melbourne, Flemington Road, Parkville, 3010, Australia; 3 Biodiversity and Geosciences, Queensland Museum, Grey Street, Brisbane, 4101, Australia

**Keywords:** alkaloid, anthelmintic, biodiscovery, bromotyrosine, 5-debromopurealidin H, *Ianthella*, NatureBank, sponge

## Abstract

In order to further expand the NatureBank open access compound library, chemical investigations of the Australian marine sponge, *Ianthella basta,* were undertaken since UHPLC–MS analysis of the extract from this sponge indicated the presence of a new alkaloid. Large-scale extraction and mass-directed isolation studies on the CH_2_Cl_2_/MeOH *I. basta* extract resulted in the purification of a new bromotyrosine-derived alkaloid, 5-debromopurealidin H (**1**), along with the known marine natural product, ianthesine E (**2**). The chemical structure of the new compound was determined following detailed spectroscopic and spectrometric data analysis. These two compounds (**1** and **2**) along with seven previously reported marine bromotyrosine alkaloids from the NatureBank open access library, which included psammaplysins F (**3**) and H (**4**), bastadins 4 (**5**), 8 (**6**) and 13 (**7**), aerothionin (**8**) and hexadellin A (**9**), were evaluated for their nematocidal activity against exsheathed third-stage larvae of *Haemonchus contortus*, a highly pathogenic parasite of ruminants. Of the nine compounds, bastadin 8 (**6**), hexadellin A (**9**) and bastadin 4 (**5**) showed inhibition towards larval motility after 72 h of exposure with IC_50_ values of 1.6 µM, 10.0 µM and 33.3 µM, respectively.

## Introduction

Marine sponges have been a significant source of unique chemistry over the past 70 years, with 11,863 sponge-derived secondary metabolites currently reported in the literature [1]. This equates to ≈30% of all marine natural products identified to date, an impressive contribution. Whilst many marine natural product chemists have shifted their attention to marine-derived microorganisms such as bacteria and fungi over the past 10 years [2], sponges continue to be a source of novel and biologically active metabolites and remain an important taxonomic phylum for the discovery of new and bioactive natural products, warranting inclusion in current and future biodiscovery programs.

Verongid sponges have been a prolific source of bioactive and novel bromotyrosine-derived alkaloids with more than 374 compounds currently reported [1]. Whilst this particular structure class includes a large number of simple bromotyrosine derivatives, more complex natural products containing spirocyclohexadienylisoxazolines, spirooxepinisoxazolines and oximes have been reported [3–4]. This class of marine alkaloids possess unique functional groups, 3D architecture and interesting pharmacology such as cytotoxicity [5–6], antibacterial [7–8], antimalarial [9], anti-HIV [10] and antifouling activities [11].

Due to our continuing interest in the identification of new secondary metabolites from Australian marine sources, in addition to further expanding the NatureBank [12] open access compound library, we have recently embarked on a biodiscovery program utilising a subset of marine specimens acquired from the Australian Institute of Marine Science (AIMS) [13] that have recently been added to the NatureBank biota repository. UHPLC–MS studies of several Verongida extracts from this new collection were analysed in order to identify samples containing abundant or potentially new natural products. A sample from the Australian marine sponge *Ianthella basta* was chosen for large-scale isolation work after analysis of the UHPLC–MS data and the MarinLit database [1] suggested the presence of a new alkaloid.

Herein, we describe the large-scale extraction, mass-directed isolation and structure elucidation of a new bromotyrosine-derived alkaloid to which we have given the trivial name 5-debromopurealidin H (**1**). The anthelmintic evaluation of this new compound, along with seven previously reported and related marine bromotyrosines sourced from the NatureBank open access compound library, is also reported.

## Results and Discussion

**Figure 1:**
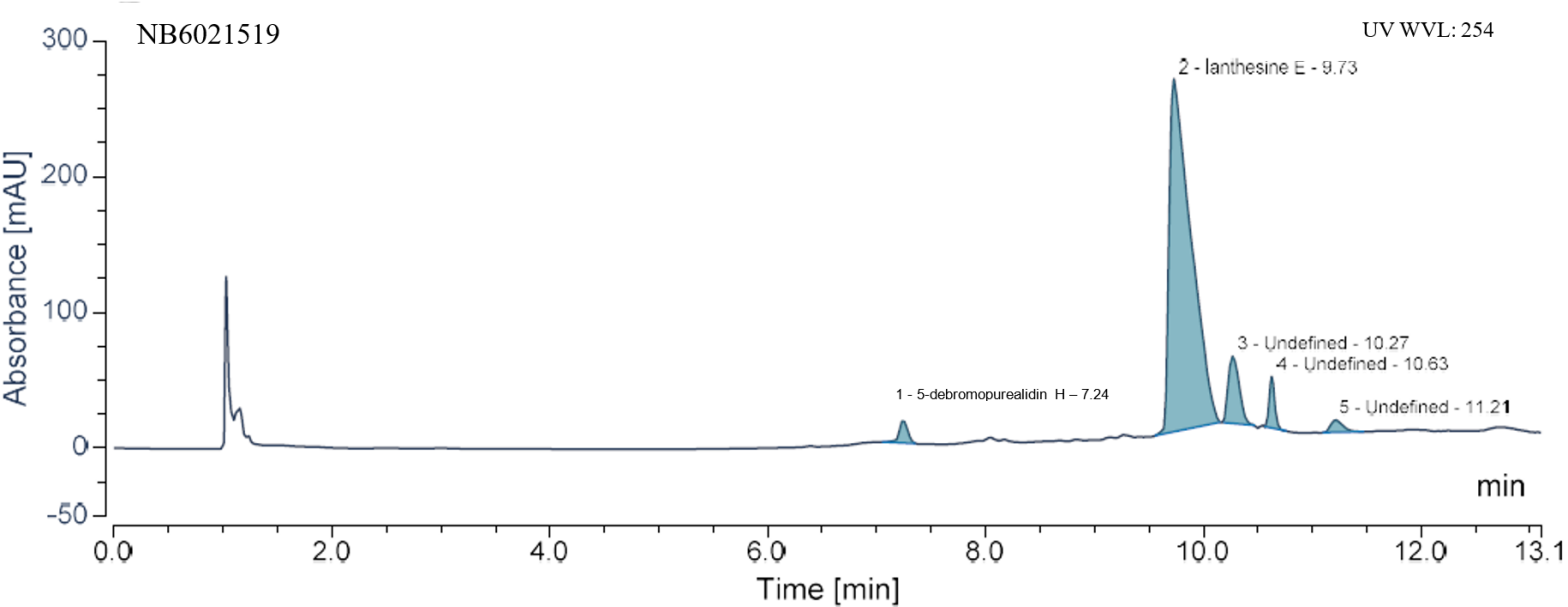
UHPLC–UV chromatogram (254 nm) of the CH_2_Cl_2_/MeOH extract of *Ianthella basta* (NB6021519); retention times for major UV peaks are indicated.

While the unique NatureBank HTS-compatible natural product screening libraries (>21K extracts and >105K fractions) [14] have been regenerated for biodiscovery collaborations with industry and academia over the past 5 years, the NatureBank open access compound library currently contains only ≈100 pure natural products. In an effort to expand this compound library, we have recently embarked on a program whereby a subset of the NatureBank marine and terrestrial extracts have been analysed by UHPLC–MS in order to determine those that contain abundant or potentially new natural products. One such example is the investigation into Verongid sponges; NatureBank holds 63 Verongida specimens that have been recently acquired from AIMS and of these, 39 have adequate material (≥10 g dry weight) for typical large-scale extraction and isolation work. Small-scale (300 mg) extraction of the abundant 39 sponge samples followed by UHPLC–MS and data analysis led to the prioritisation of several extracts for follow-up large-scale chemical investigations (see Figure S1 in Supporting Information File 1 for all UHPLC–MS chromatograms). One extract derived from the marine sponge *Ianthella basta* showed five UV active peaks (P1–5) at 254 nm in the UHPLC–MS chromatogram (Figure 1) with P1–3 and P5 displaying quasi-molecular ion clusters in the positive MS mode. Subsequent dereplication, literature and MarinLit database [1] mining tentatively identified P1 as a new monobrominated marine natural product with ions of equal relative intensity at *m*/*z* 380 and 382 [M + H]^+^. Complex ion clusters (abundance ratio: 1:4:6:4:1) at *m*/*z* 715/717/719/721/723 [M + H]^+^, 1014/1016/1018/1020/1022 [M + H]^+^ and 715/717/719/721/723 [M + H]^+^ associated with peaks two, three and five, respectively, were tentatively assigned as tetrabrominated metabolites. MS data of P4 was ambiguous and dereplication approaches were unable to successfully assign a tentative structure.

**Figure 2:**
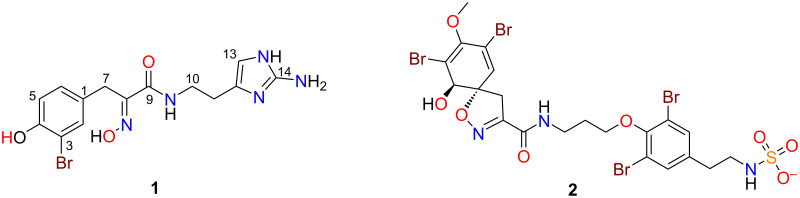
Chemical structures of 5-debromopurealidin H (**1**) and ianthesine E (**2**).

Based on these data, large-scale extraction and mass-directed isolation studies were initiated. The freeze-dried and ground specimen of *Ianthella basta* (10 g) was sequentially extracted with *n*-hexane, CH_2_Cl_2_ and MeOH, and a portion of each extract was analysed by UHPLC–MS for the ions of interest. Subsequently, the CH_2_Cl_2_ and MeOH extracts were combined (which both contained targeted ion clusters) and subjected to reversed-phase C_18_ HPLC (MeOH/H_2_O/0.1%TFA) which led to the purification of the new metabolite, 5-debromopurealidin H (**1**) as its TFA salt along with the major previously reported metabolite, ianthesine E (**2**) (Figure 2). Comparison of the 1D NMR, MS and specific rotation data for compound **2** with literature values [15] identified this metabolite as ianthesine E, which had been previously identified from a *Pseudoceratina* sp. specimen collected from the Great Barrier Reef. 5-Debromopurealidin H and ianthesine E corresponded to peaks one and two in the UHPLC–MS trace, respectively, while metabolites associated with peaks 3–5 in the UHPLC–MS were unable to be obtained in sufficient purity or quantity for structure elucidation studies. Furthermore, insufficient quantities of the raw material of the *I. basta* specimen prevent further chemical investigation studies at this point in time.

**Figure 3:**
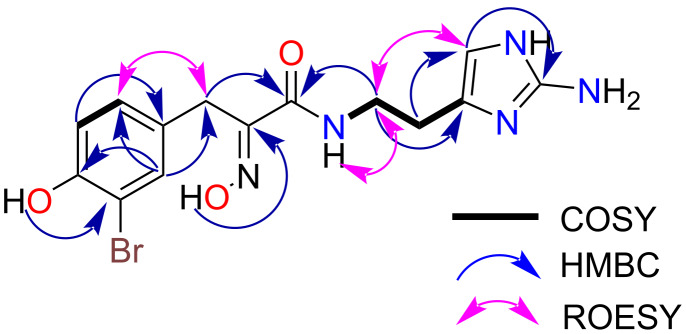
Key COSY, HMBC and ROESY correlations for 5-debromopurealidin H (**1**).

5-Debromopurealidin H (**1**) was isolated as a stable yellow film. The LRESIMS of **1** showed 1:1 ion cluster peaks at *m*/*z* 382/384 [M + H]^+^ and 380/382 [M − H]^−^, indicating the presence of one bromine atom [16]. Furthermore, a molecular formula of C_14_H_17_BrN_5_O_3_ was assigned following analysis of the 1D NMR data in conjunction with the HRESIMS ion at *m*/*z* 384.0487 [M + H]^+^. The ^1^H and COSY NMR spectra (Table 1) of **1** in DMSO-*d*_6_ revealed two distinct spin systems, which included a 1,3,4-trisubstituted aromatic ring [17–18], and an *N*-substituted ethylene system [19]. Remaining unassigned proton signals included a methylene singlet (δ_H_ 3.67), a downfield sp^2^ singlet (δ_H_ 6.55) and four exchangeable protons (δ_H_ 7.36, 8.07, 11.84, and 10.11). HMBC correlations from the methylene singlet (δ_H_ 3.67, δ_C_ 27.8) into the trisubstituted phenyl system and two downfield sp^2^ signals (δ_C_ 151.9 and 163.3), in conjunction with the HMBC data for both exchangeable signals at δ_H_ 7.36 and 11.84, and methylene moiety (δ_H_ 3.35, δ_C_ 39.4) to the sp^2^ signals (δ_C_ 151.9 and 163.3) allowed a *N*-oxime substructure to be assigned (see Figure 3).

**Table 1:** NMR data for the TFA salt of 5-debromopurealidin H (**1**) in DMSO-*d*_6_.^a^

position	δ_C_, type	δ_H_, mult (*J* in Hz)	COSY	HMBC	ROESY

1	128.8, C				
2	132.8, CH	7.27, d (2.1)	6	3, 4, 6, 7	7
3	108.9, C				
4	152.4, C				
4-OH		10.11, brs		3, 4^b^	
5	116.2, CH	6.83, d (8.3)	6	1, 3, 4^b^	6
6	129.1, CH	6.99, dd (2.1, 8.3)	2, 5	2, 4, 7	5, 7
7	27.8, CH_2_	3.67, s		2, 6, 8, 9	2, 6
8	151.9, C				
8-NOH		11.84, s		8	
9	163.3, C				
9-NH		8.07, t (6.0)	10	9^b^	10
10	37.2, CH_2_	3.35, dt (6.0, 6.9)	9-NH, 11	9, 12	9-NH, 11, 13
11	24.5, CH_2_	2.59, t (6.9)	10, 13	12, 13	10, 13
12	124.4, C				
13	109.3, CH	6.55, s	11	12, 14	10, 11
14	146.8, C				
14-NH_2_		7.36, brs			

^a1^H, 800 MHz; ^13^C, 200 MHz; ^b^weak correlation.

MarinLit database mining using this fragment readily identified that compound **1** belonged to the purealidin structure class, the majority of which all contain a terminal imidazole system. HMBC data from the one of the methylene protons (δ_H_ 2.59) that constituted the -NHCH_2_CH_2_- spin system to the imidazole carbons at δ_C_ 124.4 and 109.3 linked these fragments, while a strong HMBC correlation from the imidazole proton at δ_H_ 6.55 to the downfield carbon at δ_C_ 146.8 indicated a 2-amino substituted histamine moiety [19]. Finally, the unassigned exchangeable protons at δ_H_ 10.11 and 7.36, were assigned to phenol (4-OH) and amino groups (14-NH_2_), respectively, following comparison of NMR data with related previously reported purealidins [19]. While no HMBC correlations were identified for these exchangeable proton signals, the presence of a phenol was further supported by a bathochromic shift that was seen in the UV spectrum of **1** upon addition of base [20]. The bromine atom and phenol hydroxy group were positioned at C-3 and C-4 of the aromatic ring, respectively, based on NMR chemical shift data comparison with related marine natural products [19]. The *E* configuration for the oxime in **1** was assigned by the diagnostic carbon chemical shifts of the benzylic methylene (C-7, δ_C_ 27.8) [21]. Thus, the chemical structure **1** was assigned as 5-debromopurealidin H.

**Figure 4:**
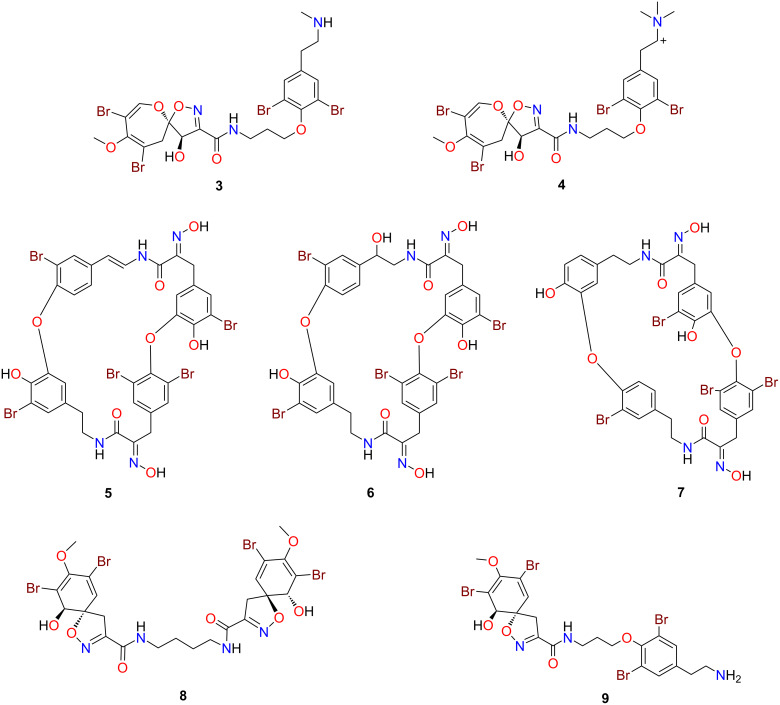
Chemical structures of the NatureBank bromotyrosine derivatives: psammaplysins F (**3**) and H (**4**), bastadins 4 (**5**), 8 (**6**) and 13 (**7**), aerothionin (**8**) and hexadellin A (**9**).

Due to our interest in discovering new anthelmintics from marine sources [22–26], we decided to test these compounds for their nematocidal activity against *Haemonchus contortus*, a highly pathogenic parasitic nematode of ruminants [27]. A structure-based search of the ≈100 NatureBank pure compound library identified seven previously reported bromotyrosines that were available in quantities that would enable in vitro anthelmintic evaluations. These compounds (Figure 4) included psammaplysins F (**3**) and H (**4**), bastadins 4 (**5**), 8 (**6**) and 13 (**7**), aerothionin (**8**) and hexadellin A (**9**). The extraction and isolation of these known compounds has been previously reported elsewhere [11,28–30]

**Figure 5:**
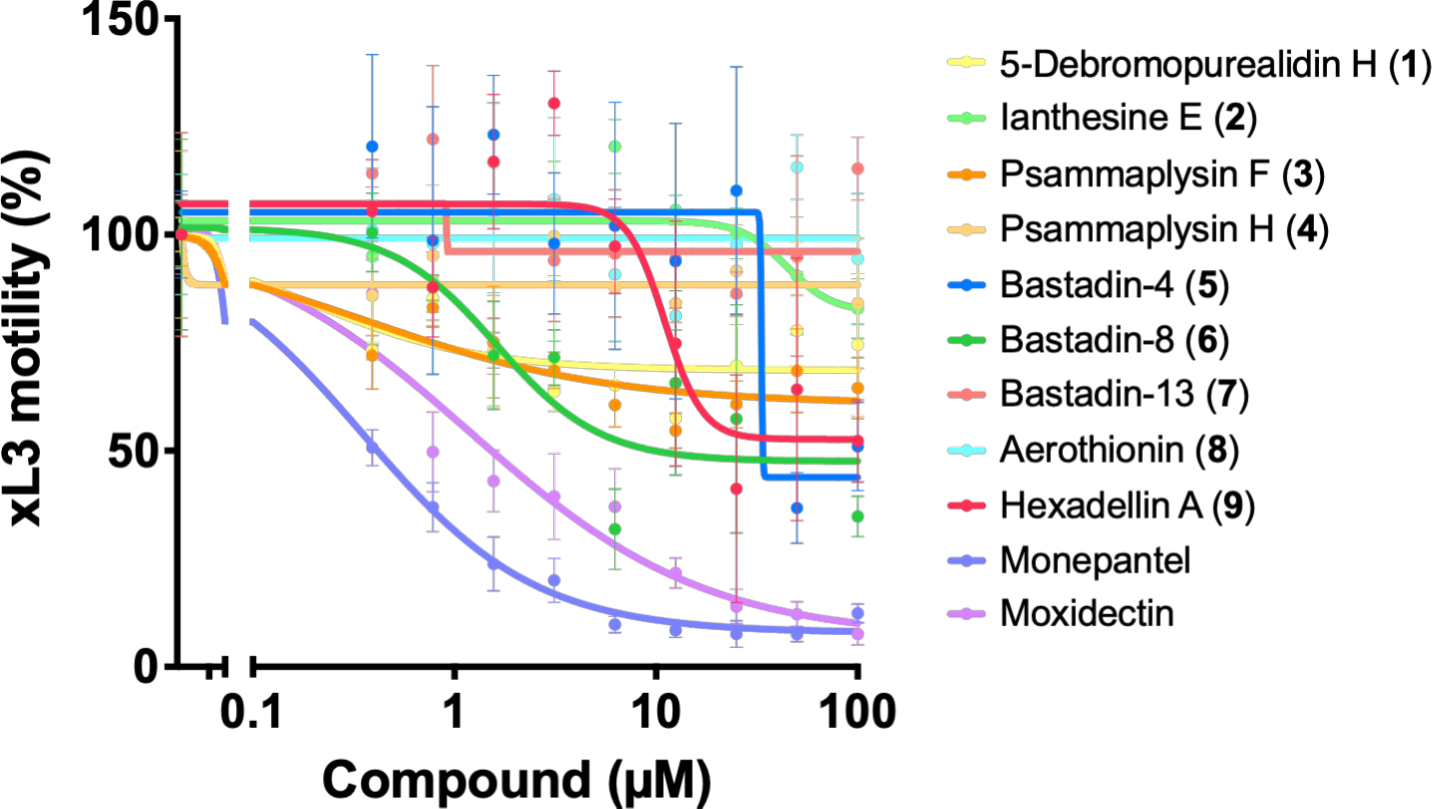
Dose-response assessment of in vitro activity of compounds **1**–**9** against exsheathed third-stage larvae (xL3) of *Haemonchus contortus*. The potency of compounds **1**–**9** on the larval motility was assessed after 72 h of exposure by establishing potency (IC_50_). Two positive controls, monepantel and moxidectin, were included as active compound references. Data points represent three independent experiments conducted in triplicate: the mean ± standard error of the mean (SEM).

Compounds **1**−**9** were assessed for their activities and potencies in a dose-response assay by measuring the inhibition level on larval motility after 72 h of exposure (Figure 5). Compound **6** was the most potent compound at inhibiting exsheathed third-stage larvae (xL3) motility after 72 h, with an IC_50_ value of 1.6 ± 0.4 µM (65.2 ± 4.7% inhibition at 100 µM). Two other compounds showed relatively high potency: compounds **9** (IC_50_ = 10.0 ± 1.9 µM; 47.7 ± 9.5% inhibition at 100 µM) and **5** (IC_50_ = 33.3 ± 4.7 µM; 49.0 ± 10.2% inhibition at 100 µM). Although not as potent, compounds **1** and **3** were active against xL3s and reduced motility at 100 µM (30.4 ± 16.4% and 35.5 ± 7.1% inhibitions, respectively). Compounds **4**, **7** and **8** had no effects on xL3 after 72 h of exposure.

None of the bromotyrosines tested in these studies had previously been tested for anthelmintic activity, however, several of them have been shown to display a variety of biological activities. For example, some ianthesines have been shown to display Na,K-ATPase inhibitory activity [31] and antimicrobial activity [32]; ianthesine E has rarely been tested for bioactivity, however, some weak binding has been identified for this molecule against human adenosine A1 receptor 19, along with some weak cytotoxicity towards HeLa cells [15]. Limited testing has also been conducted on the purealidin bromotyrosine structure class, however, quorum sensing inhibition and antifouling activities against several strains of bacteria and microalgae appears in the literature [33–34], while HIV-1 replication inhibition and anti-*Leishmania* and *Plasmodium* activity has also been recorded [35–36]. The psammaplysin structure class has also had antimalarial [28], cytotoxicity [37] and antimicrobial data reported, albeit with low to moderate potencies [7]. More recently psammaplysin F and several semi-synthetic analogues have been shown to cause loss of mitochondrial membrane potential, fragmentation of the mitochondrial tubular network, chromosome misalignment, and cell cycle arrest in mitosis in LNCaP prostate cancer cells [38]. The bastadin structure class is well-documented within the literature for their cytotoxic activity [37,39–41], with both bastadins 4 and 8 exhibiting in vitro cytotoxicity against leukemia cell line L-1210 (ED_50_ 5 µg/mL) [39]. Aerothionin has been previously shown to exhibit antimycobacterial activity against monoresistant variants of *Mycobacterium tuberculosis* H37Rv, leading to further reported activity in various *M. tuberculosis* clinical isolates as well as non-tuberculosis mycobacteria [42]; while antitumour [43] and antifouling [44] activities have also been published.

## Conclusion

In summary, we report here the UHPLC–MS profiling of 39 Verongida sponge extracts from NatureBank, which led to large-scale extraction and mass-directed isolation studies on an *Ianthella basta* specimen and resulted in the purification of a new alkaloid that we named, 5-debromopurealidin H (**1**). Testing of this bromotyrosine-derived natural product along with several related marine metabolites in an in vitro anthelmintic assay showed that five out of nine metabolites, including 5-debromopurealidin H (**1**), had activity against larvae of the parasitic nematode, *H. contortus*. Definitive structure assignments for the unidentified metabolites from the *I. basta* extract would require recollection of this sponge, which is currently not feasible. Additional Verongida extracts used in the UHPLC–MS profiling reported here will be targeted for follow-up large-scale chemical investigations in the future.

## Experimental

### General experimental procedures

Specific rotations were recorded using a JASCO P-1020 polarimeter. IR data were acquired on a Universal Attenuated Total Reflectance (UATR) Two attachment on a PerkinElmer spectrometer. UV data was recorded on a JASCO V-650 UV–vis spectrophotometer. NMR spectra were recorded at 25 °C on a Bruker AVANCE III HD 500 or 800 MHz NMR spectrometer equipped with a cryoprobe. The ^1^H and ^13^C chemical shifts were referenced to the solvent peak for DMSO-*d*_6_ at δ_H_ 2.50 and δ_C_ 39.52, respectively. The UHPLC–MS data were recorded on an Ultimate 3000 RS UHPLC coupled to a Thermo Fisher Scientific ISQEC single quadruple ESI mass spectrometer using an analytical Thermo Scientific Accucore C_18_ (2.6 μm, 80 Å, 150 × 2.1 mm). HRESIMS data was acquired on a Bruker maXis II ETD ESI-qTOF. Phenomenex Strata solid phase extraction (SPE) cartridges (3 cc, polypropylene, single fritted) were used for the small-scale marine sponge extractions. GRACE Davisil (35–70 µm, 60 Å) C_18_-bonded silica was used for pre-absorption work before reversed-phase (RP) HPLC. The preabsorbed material was subsequently packed into an Alltech stainless steel guard cartridge (10 × 30 mm) then attached to a HPLC column prior to fractionation. A Waters 600 pump fitted with a Waters 996 photodiode array detector fitted with a Gilson 717-plus autosampler were used for RP-HPLC separations. Frozen marine sponges from AIMS were dried using a Dynamic FD12 freeze dryer and ground using a Fritsch Universal Cutting Mill Pulverisette 19, or by hand using a granite mortar and pestle. For large-scale extraction work, the ground sponge material was extracted at room temperature using an Edwards Instrument Company Bio-line orbital shaker set to 200 rpm. Solvents were removed from crude marine extracts with a Buchi R-144 rotary evaporator and from HPLC fractions using a GeneVac XL4 centrifugal evaporator. All solvents used for chromatography, UV, MS and [α]_D_ were Honeywell Burdick & Jackson or Lab-Scan HPLC grade. H_2_O was filtered using a Sartorius Stedium Arium^®^ Pro VF ultrapure water system.

### Sponge material

The 39 Verongida sponge samples were obtained from the NatureBank biota library housed at the Griffith Institute for Drug Discovery, Griffith University, Australia [12]. All samples were collected and taxonomically identified by AIMS [13]. All sponge samples received from AIMS were freeze-dried, ground and stored at room temperature prior to extraction. The specimen of *Ianthella basta* (NB6021519; phylum Porifera, class Demospongiae, order Verongida, family Ianthellidae) used in the UHPLC–MS and subsequent large-scale extraction and isolation investigations was collected on the 17 September 2012 from the Timor Sea in the north-western area of the Bonaparte Basin within the Oceanic Shoals Marine Park, Australia. A voucher specimen (G337610) has been lodged at the Queensland Museum, South Brisbane, Queensland, Australia.

### Preparation of sponge extracts for UHPLC–MS analysis

Each of the Verongida samples (300 mg) was packed into an SPE cartridge and sequentially extracted with CH_2_Cl_2_ (7 mL) and MeOH (7 mL) then combined. The CH_2_Cl_2_/MeOH extract was prepared for UHPLC–MS analysis using MeOH to generate a stock solution, which had a final concentration of 1 mg/mL (minimum stock solution volume = 0.5 mL).

### UHPLC–MS analysis

All extracts (1 mg/mL) were subjected to UHPLC–MS profiling using an Thermo Scientific Accucore column with an injection volume of 10 µL. Isocratic conditions of 10% MeOH/90% H_2_O (0.1% formic acid) were employed for the first minute, followed by a linear gradient to 100% MeOH (0.1% formic acid) over 8 min followed by a 1.5 min isocratic elution of 100% MeOH (0.1% formic acid) all at a flow rate of 0.3 mL/min. UHPLC–MS data was analysed using Thermo Scientific Dionex Chromeleon 7 software (Version 7.2.10) at a wavelength of 254 nm.

### Large-scale extraction and mass-directed isolation of *Ianthella basta*

In a manner similar to Yang et al. [9], the freeze-dried and ground specimen of *Ianthella basta* (NB6021519; 10 g) was extracted sequentially with *n*-hexane (250 mL), CH_2_Cl_2_ (250 mL) and MeOH (250 mL × 2). The *n*-hexane extract was discarded (as it contained only highly lipophilic material) and the CH_2_Cl_2_ and MeOH extracts were combined to produce a brown extract (2.36 g). This extract was pre-absorbed to C_18_-bonded silica (1 g) in 200 mg portions, packed into a stainless-steel guard cartridge (10 × 30 mm) and subjected to C_18_ semipreparative HPLC. Isocratic HPLC conditions of 90% H_2_O (0.1% TFA)/10% MeOH (0.1% TFA) were initially employed for the first 10 min, then a linear gradient to 100% MeOH (0.1% TFA) was run over 40 min, followed by isocratic conditions of 100% MeOH (0.1% TFA) for a further 10 min, all at a flow rate of 9 mL/min. Sixty 1 min fractions were collected from time 0 to 60 min to afford 5-debromopurealidin H (**1**, 11.1 mg, *t*_R_ 32–34 min, 0.111% dry wt), and the known alkaloid ianthesine E (**2**, 161.2 mg, *t*_R_ 40–41 min, 1.612% dry wt).

**5-Debromopurealidin H (1):** Yellow film; UV (MeOH) λ_max_ (log ε) 283 (3.02) nm; UV (MeOH + NaOH) λ_max_ (log ε) 236 (3.53), 300 (2.94) nm; IR (UATR) v_max_ 3357, 1678, 1429, 1204, 1140 cm^−1^; ^1^H and ^13^C NMR data, Table 1; (+)-LRESIMS *m*/z 382/384 (1:1) [M + H]^+^; (−)-LRESIMS *m*/z 380/382 (1:1) [M − H]^−^; (+)-HRESIMS *m*/*z* 382.0507 [M + H]^+^ (calcd for C_14_H_17_^79^BrN_5_O_3_, 382.0509).

**Ianthesine E (2):** Yellow film; 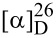 + 76.3 (*c* 0.4 in MeOH), lit. 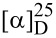 + 50.6 (*c* 0.3 in MeOH) [15]; (+)-LRESIMS *m*/*z* 714/716/718/720/722 (1:4:6:4:1) [M + H]^+^, (−)-LRESIMS *m*/z 792/794/796/798/800 (1:4:6:4:1) [M − H]^−^.

### Preparation of nematode larvae for bioassays

*Haemonchus contortus* (Haecon-5 strain) larvae (L3s) were produced and stored using an established protocol [45] – approved by the animal ethics committee of the University of Melbourne (permit no. 1714374). For use in the assay, L3s were exsheathed and sterilized by incubation in 0.15% (v/v) sodium hypochlorite (NaClO) at 38 °C for 20 min [45] and then washed five times in sterile saline by centrifugation at 500*g* (5 min) at room temperature (22–24 °C). After the last wash, exsheathed L3s (xL3s) were suspended in lysogeny broth (LB) containing 100 IU/mL of penicillin, 100 µg/mL of streptomycin and 0.25 µg/mL of amphotericin B – designated LB*.

### Bioassay for the evaluation of anthelmintic activity of purified compounds

The bromotyrosine compounds (**1**–**9**), were individually tested for their anthelmintic effect on larvae (xL3s) of *H. contortus* using an established bioassay [45]. Each assay was performed in triplicate on three different days. In brief, compounds were serially diluted in 50 μL of LB* (18-points, 2-fold dilution, 100 µM to 0.76 nM) and dispensed into the wells of sterile 96-well flat-bottom microtitre plates containing 300 xL3s; with six wells with no compound (LB* + 0.25% DMSO; negative control). A plate containing monepantel or moxidectin (positive control compounds) was serially diluted and prepared in the same manner. The motility of larvae was measured at 72 h.

## Supporting Information

File 1 UHPLC–UV chromatograms, characterisation data and copies of spectra for 5-debromopurealidin H (**1**).
