# Supplementary material for: Using UHPLC–MS profiling for the discovery of new sponge-derived metabolites and anthelmintic screening of the NatureBank bromotyrosine library
Source: Beilstein J Org Chem. 2022 Nov 15;18:1544–52. doi: 10.3762/bjoc.18.164 (PMC9679598; doi:10.3762/bjoc.18.164)
Supplement: File 1 — UHPLC–UV chromatograms, characterisation data and copies of spectra for 5-debromopurealidin H (1). [file Beilstein_J_Org_Chem-18-1544-s001.pdf]

BEILSTEIN JOURNAL OF ORGANIC CHEMISTRY

Supporting Information

for

Using UHPLC–MS profiling for the discovery of new sponge-derived metabolites and anthelmintic screening of the NatureBank bromotyrosine library

Sasha Hayes, Aya C. Taki, Kah Yean Lum, Joseph J. Byrne, Merrick G. Ekins, Robin B. Gasser and Rohan A. Davis

Beilstein J. Org. Chem. **2022,** *18,* 1544–1552. doi:10.3762/bjoc.18.164

UHPLC–UV chromatograms, characterisation data and copies of spectra for 5-debromopurealidin H (1)

License and Terms: This is a supporting information file under the terms of the Creative Commons Attribution License (https://creativecommons.org/licenses/by/4.0). Please note that the reuse, redistribution and reproduction in particular requires that the author(s) and source are credited and that individual graphics may be subject to special legal provisions.
The license is subject to the *Beilstein Journal of Organic Chemistry* terms and conditions: (https://www.beilstein-journals.org/bjoc/terms)

Table of contents

Figure S1: UHPLC chromatograms (254 nm) of 39 CH_2Cl_2/MeOH extracts of NatureBank Verongida sponges
Figure S2: ^{1}H NMR (800 MHz) spectrum of 5-debromopurealidin H (**1**) in DMSO-d_6
Figure S3: ^{13}C NMR (200 MHz) spectrum of 5-debromopurealidin H (**1**) in DMSO-d_6
Figure S4: HSQC spectrum of 5-debromopurealidin H (**1**) in DMSO-d_6
Figure S5: COSY spectrum of 5-debromopurealidin H (**1**) in DMSO-d_6
Figure S6: HMBC spectrum of 5-debromopurealidin H (**1**) in DMSO-d_6
Figure S7: ROESY spectrum of 5-debromopurealidin H (**1**) in DMSO-d_6
Figure S8: HRESIMS of 5-debromopurealidin H (**1**)

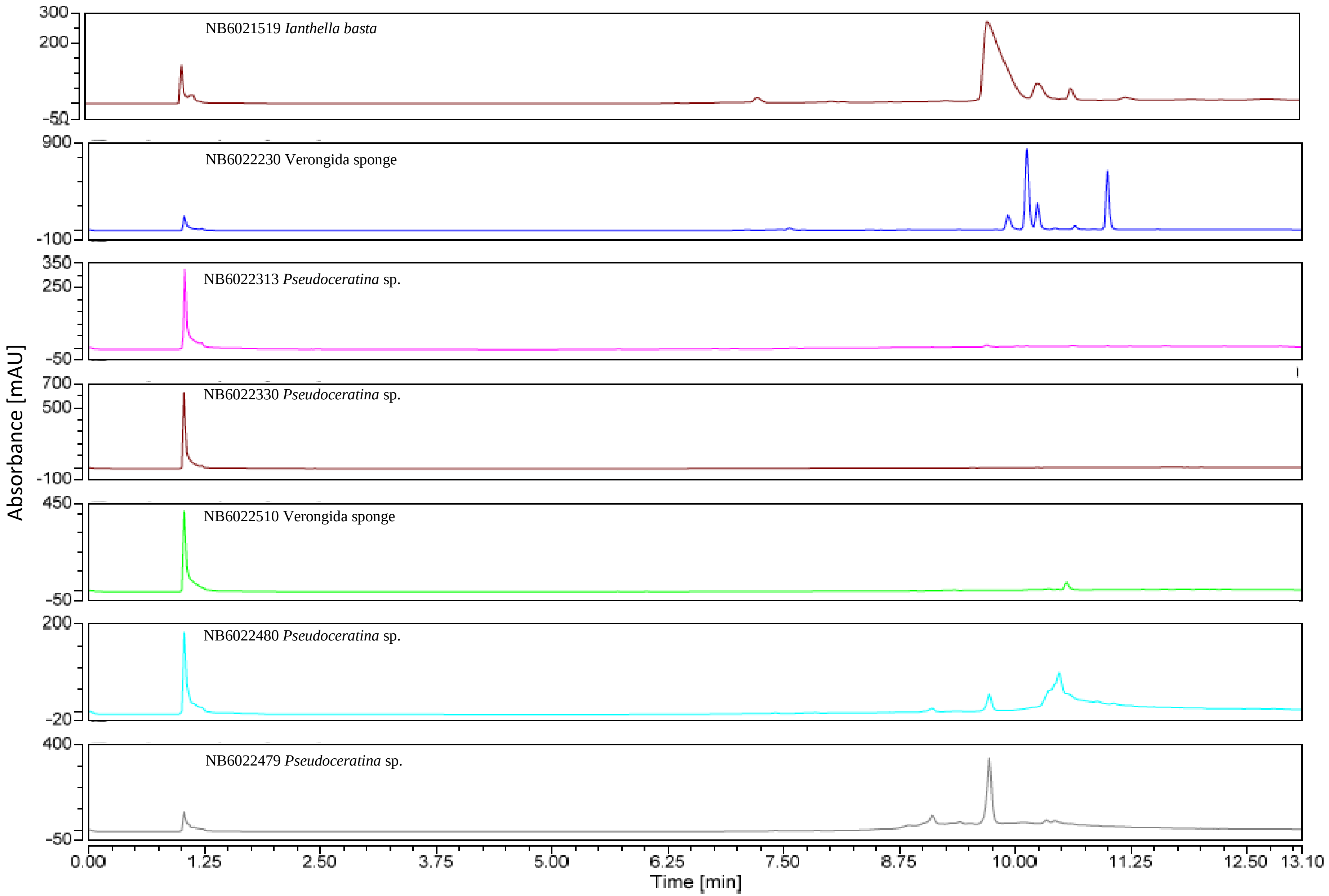

Figure S1: UHPLC chromatograms (254 nm) of 39 CH_2Cl_2/MeOH extracts of NatureBank Verongida sponges

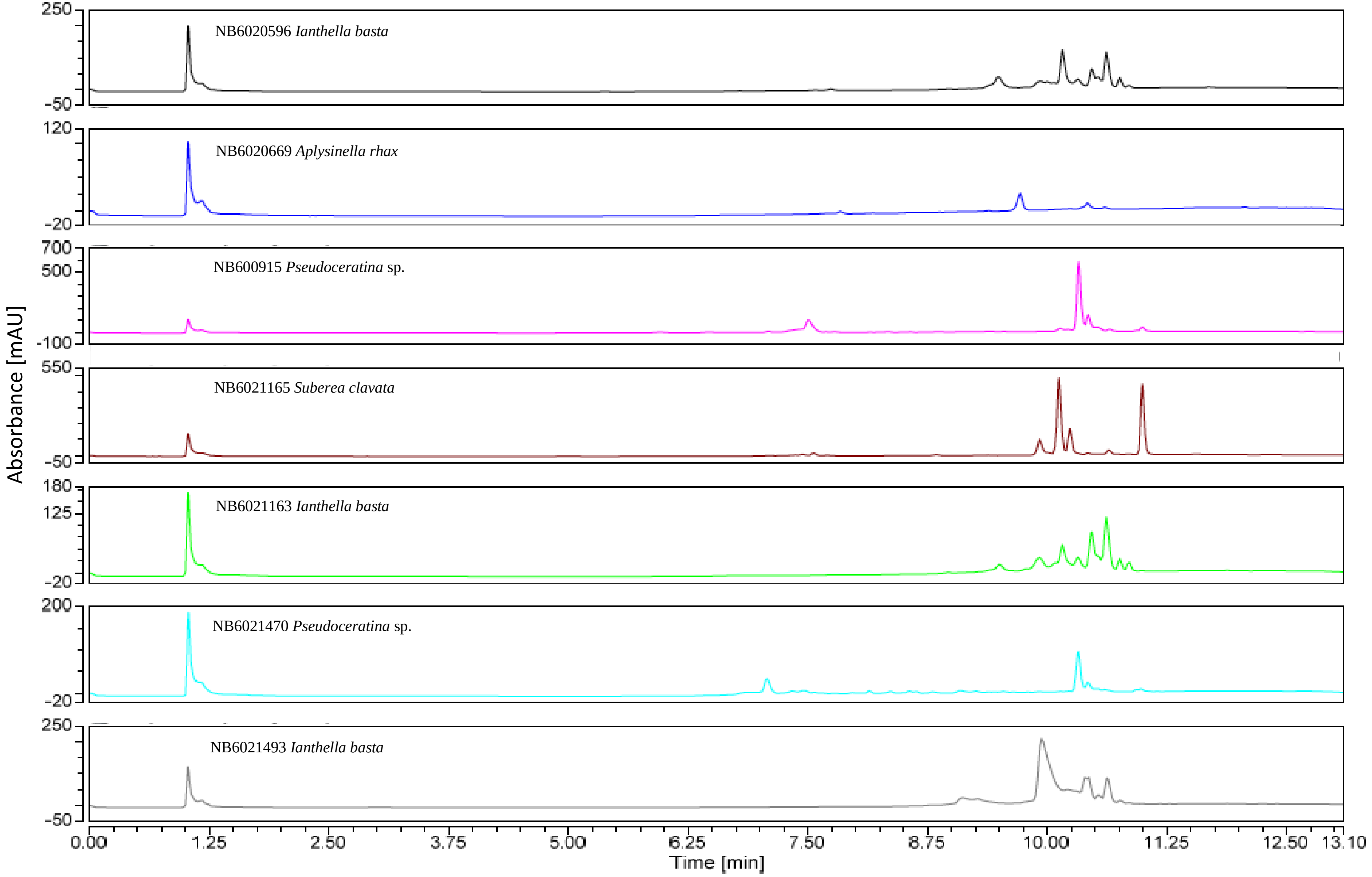

Figure S1: UHPLC chromatograms (254 nm) of 39 CH_2Cl_2/MeOH extracts of NatureBank Verongida sponges

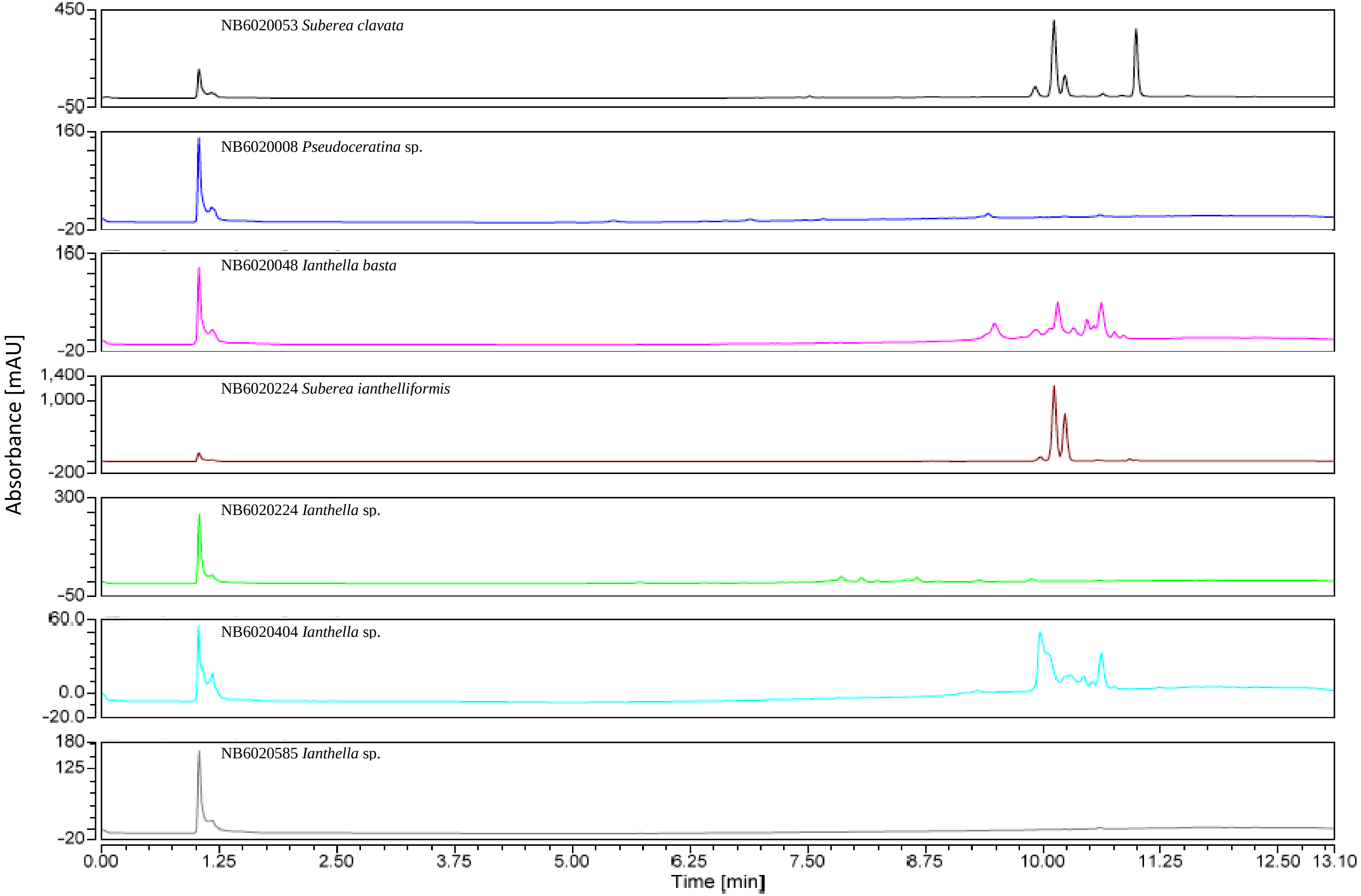

Figure S1: UHPLC chromatograms (254 nm) of 39 CH_2Cl_2/MeOH extracts of NatureBank Verongida sponges

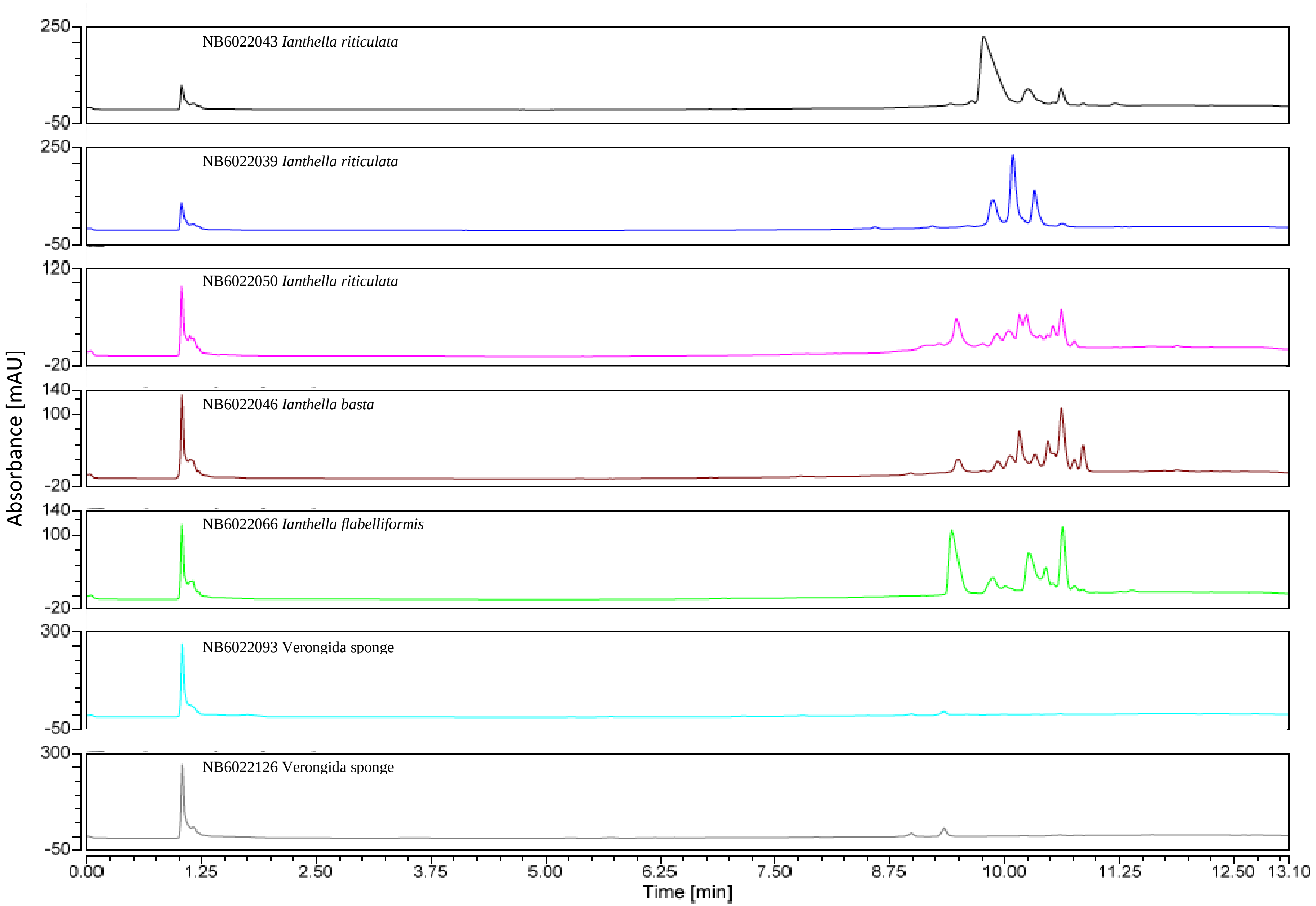

Figure S1: UHPLC chromatograms (254 nm) of 39 CH_2Cl_2/MeOH extracts of NatureBank Verongida sponges

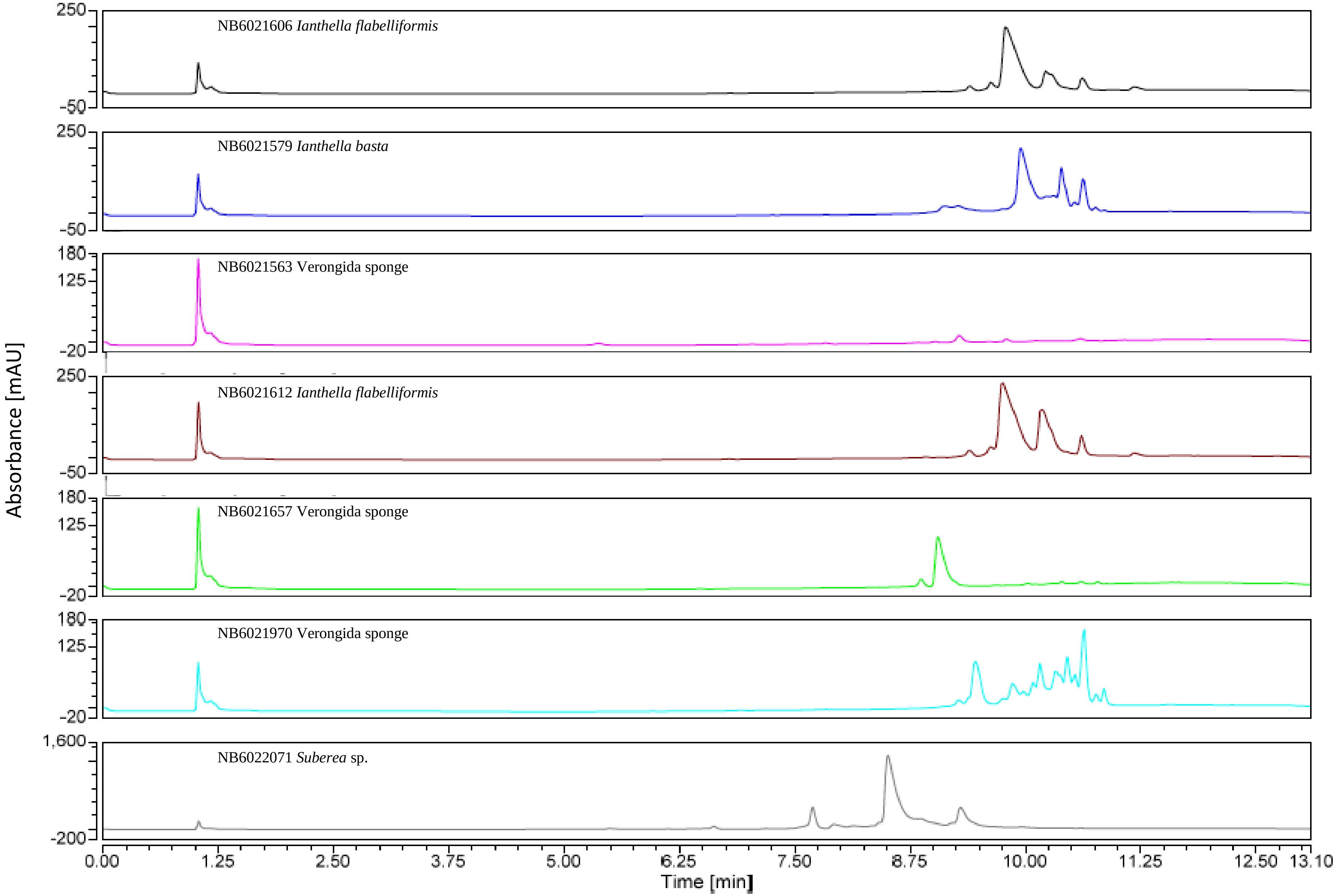

Figure S1: UHPLC chromatograms (254 nm) of 39 CH_2Cl_2/MeOH extracts of NatureBank Verongida sponges

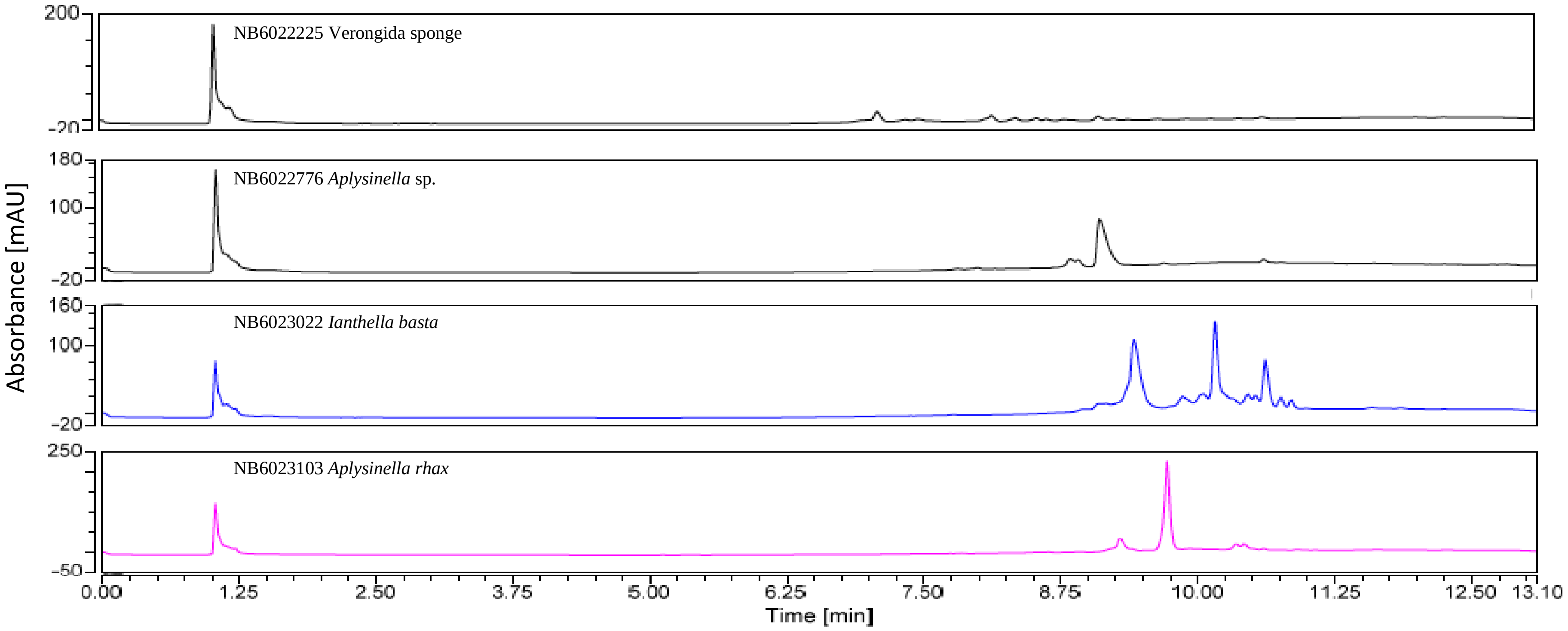

Figure S1: UHPLC chromatograms (254 nm) of 39 CH_2Cl_2/MeOH extracts of NatureBank Verongida sponges

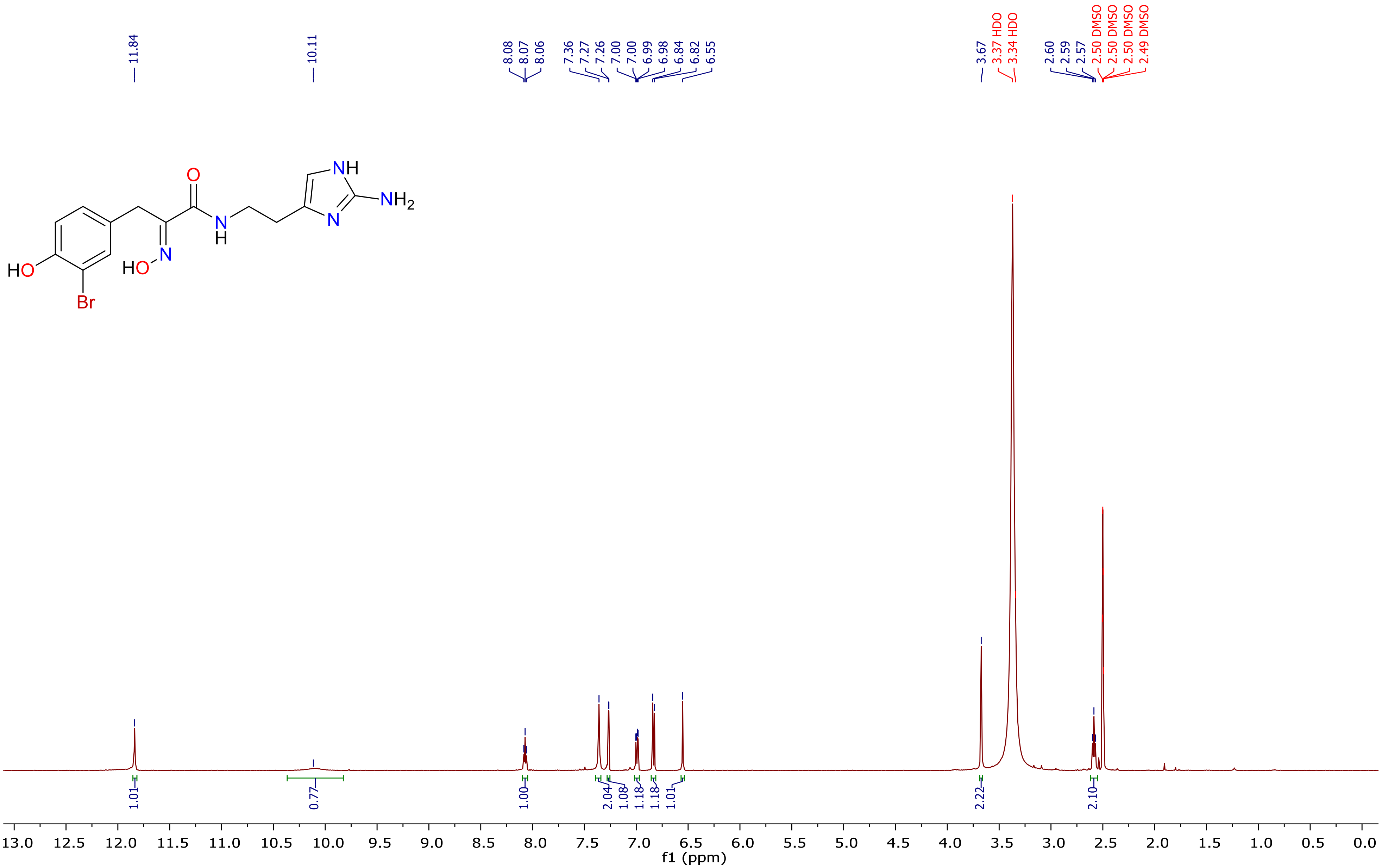

Figure S2: ^{1}H NMR (800 MHz) spectrum of 5-debromopurealidin H (**1**) in DMSO-d_6

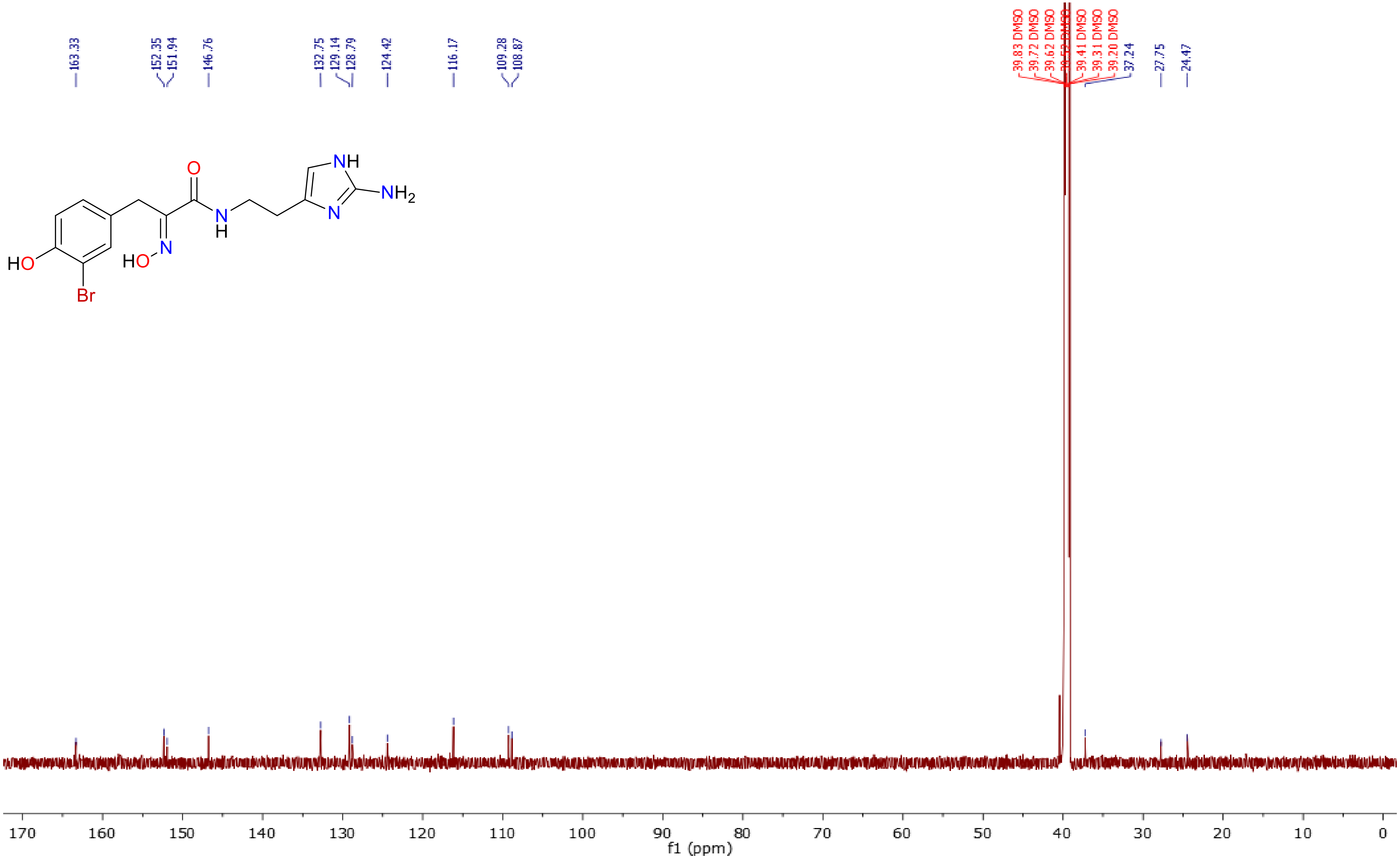

Figure S3: ^{13}C NMR (200 MHz) spectrum of 5-debromopurealidin H (**1**) in DMSO-d_6

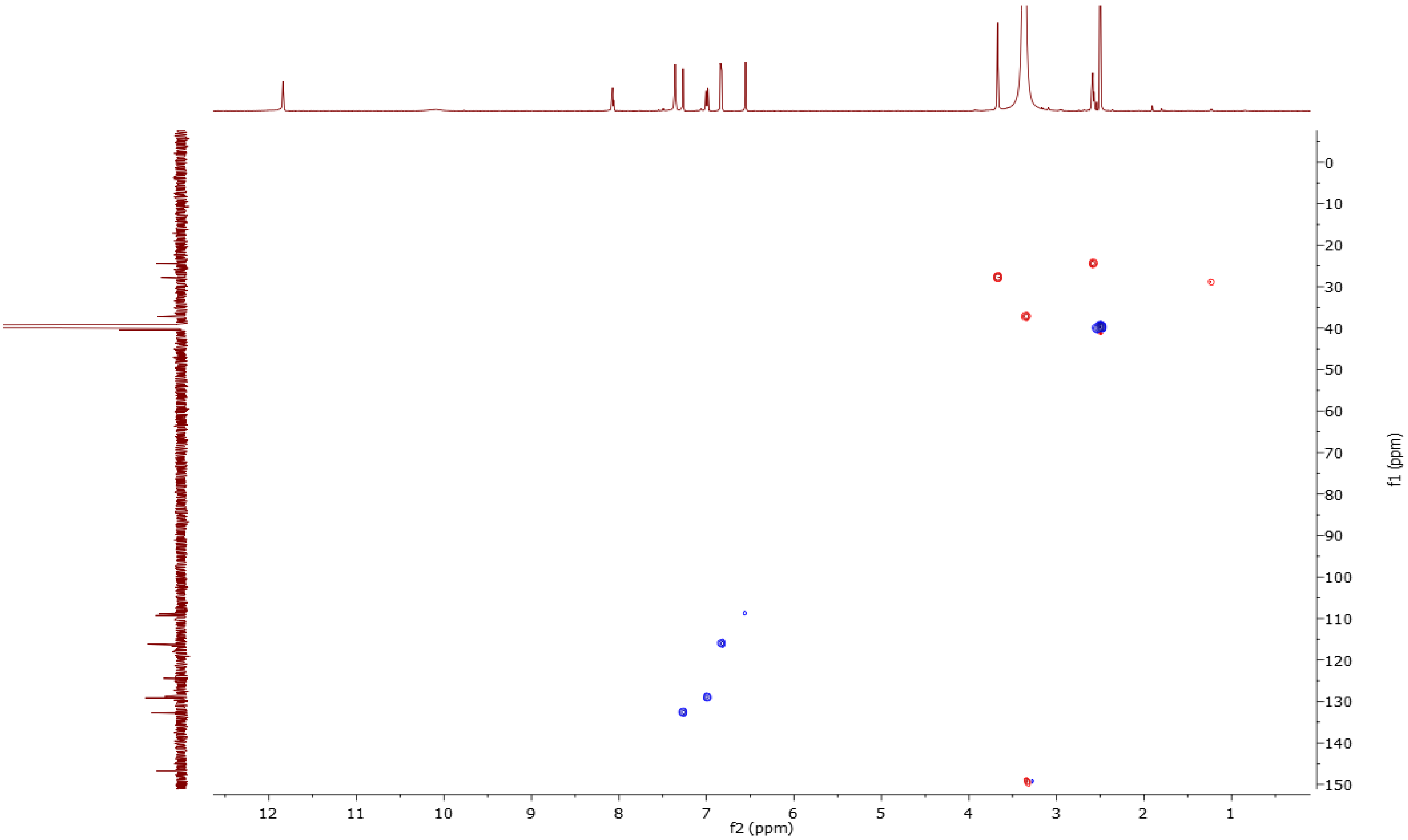

Figure S4: HSQC spectrum of 5-debromopurealidin H (**1**) in DMSO-d_6

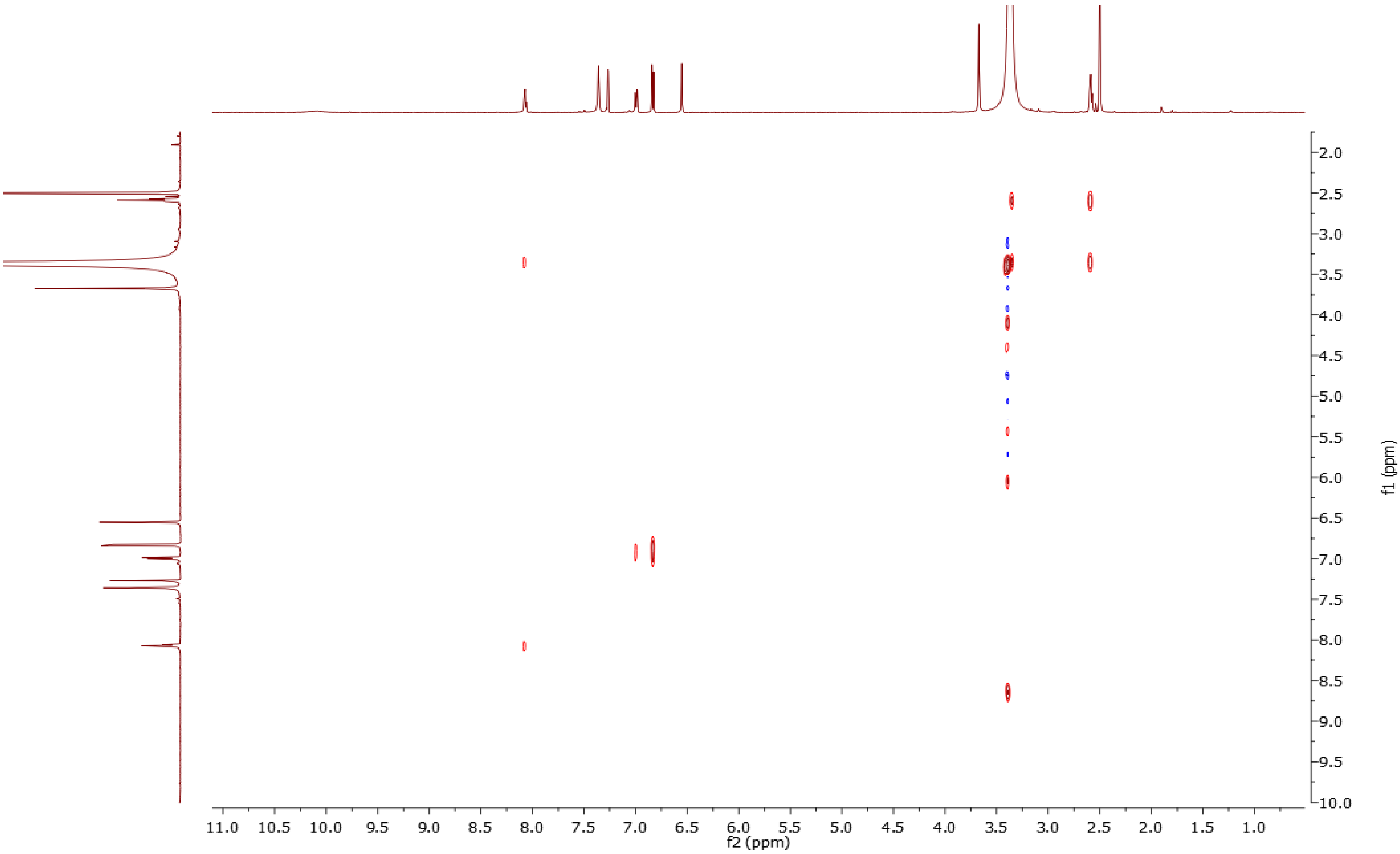

Figure S5: COSY spectrum of 5-debromopurealidin H (**1**) in DMSO-d_6

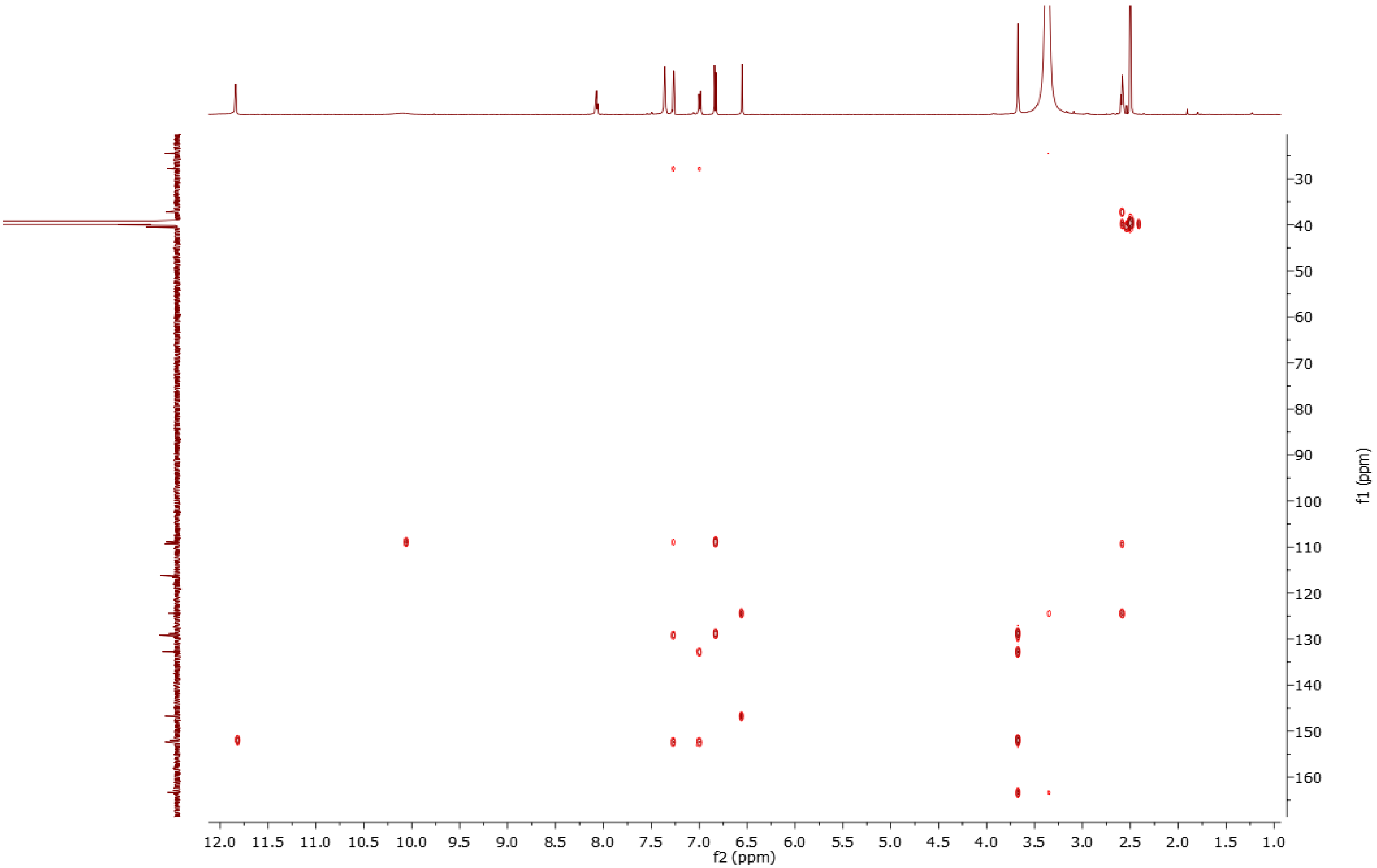

Figure S6: HMBC spectrum of 5-debromopurealidin H (**1**) in DMSO-d_6

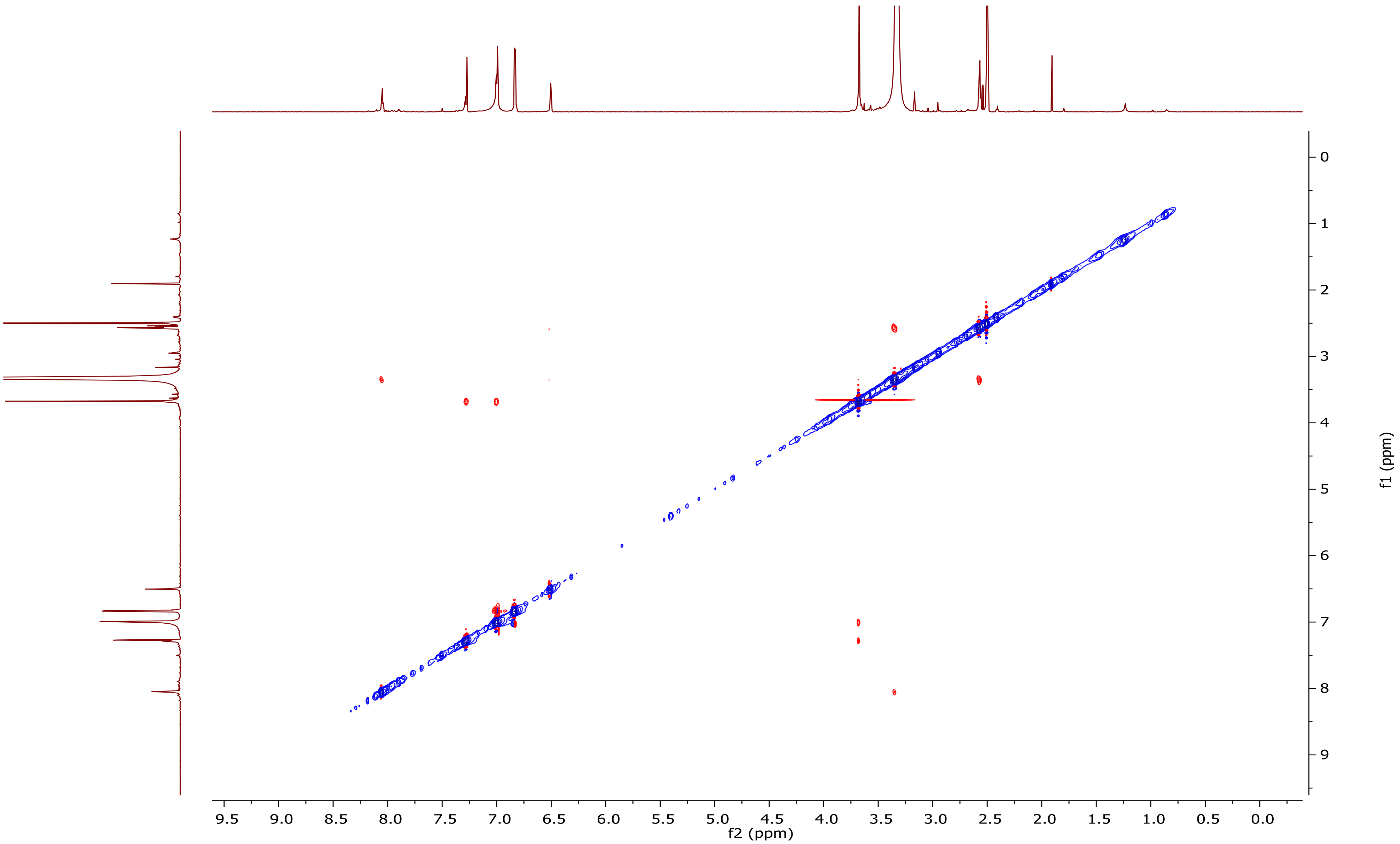

Figure S7: ROESY spectrum of 5-debromopurealidin H (**1**) in DMSO-*d*6

Griffith UNIVERSITY

High Resolution Mass Spectrum

Analysis Info

Analysis Name	D:\Data\KahYean\20200508\NBC110000001.d
Method	DirectInfusion_2018_pos.m
Sample Name	NBC110
Comment	

Acquisition Date	5/8/2020 10:30:32 AM
Instrument	maXis II ETD 1823391.22321

+MS, 0.1min #4

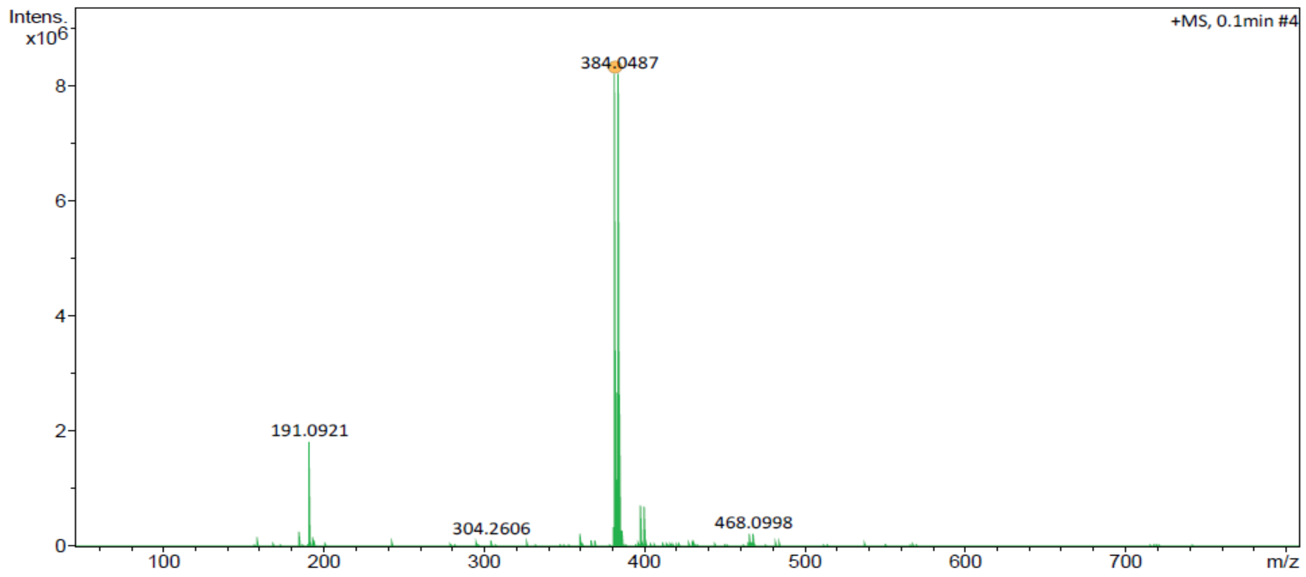

Meas. m/z	#	Ion Formula	m/z	err [ppm]	mSigma	# mSigma	Score	rdb	e⁻ Conf	N-Rule
382.0507	1	C14H17BrN5O3	382.0509	0.5	96.2	1	100.00	8.5	even	ok

Figure S8: HRESIMS of 5-debromopurealidin H (**1**)
